# *Trichosporon inkin* meningitis in Northeast Brazil: first case report and review of the literature

**DOI:** 10.1186/s12879-018-3363-7

**Published:** 2018-09-18

**Authors:** Eveline Pipolo Milan, Walicyranison Plinio Silva-Rocha, Jéssica Jacinto Salviano de Almeida, Tatiane Uetti Gomes Fernandes, André Luciano de Araújo Prudente, Matheus Firmino de Azevedo, Elaine Cristina Francisco, Analy Salles de Azevedo Melo, Arnaldo Lopes Colombo, Guilherme Maranhão Chaves

**Affiliations:** 1 Departamento de Infectologia, Universidade Federal do Rio Grande do Norte, Natal, Rio Grande do Norte Brazil; 2 Centro de Ciências da Saúde, Laboratório de Micologia Médica e Molecular, Departamento de Análises Clínicas e Toxicológicas, Universidade Federal do Rio Grande do Norte, Rua Gal. Gustavo Cordeiro de Faria S/N, Petrópolis, Natal, Rio Grande do Norte Brazil; 3 Laboratório Especial de Micologia, Disciplina de Infectologia, Universidade Federal de São Paulo, São Paulo, Brazil

**Keywords:** Invasive Trichosporonosis, Meningoencephalitis, *Trichosporon inkin*, Virulence factors, Antifungal susceptibility testing, Northeast Brazil

## Abstract

**Background:**

*Trichosporon* species may colonize the skin, respiratory tract and gastrointestinal tract of human beings. The yeast is recognized as etiological agent of *white piedra*, a superficial mycosis. Nevertheless, immunocompromised hosts may develop invasive Trichosporonosis. Central nervous system trichosporonosis is a very rare clinical manifestation. In fact, only a few cases have been published in the literature and none of them was caused by *Trichosporon inkin*.

**Case presentation:**

Here we report the first clinical case of meningoencephalitis due to this species in a female previously healthy patient under corticosteroids and antibiotics therapy for several months. She was submitted to an invasive procedure to remove a left sided acoustic neuroma and further developed a cerebrospinal fistula. After some days of the procedure, she presented a predominantly and intensive occipital holocranial headache, followed by vomiting, hyporexia, weight loss, asthenia, irritability, difficulty to concentrate and rotator vertigo. The patient further developed a cerebrospinal fistula in the occipital region and was submitted to a surgical correction. After several months of clinical interventions, she was diagnosed with CNS Trichosporonosis, after Magnetic Resonance Imaging and positive microbiological cultures obtained within two different occasions (2 weeks apart). Despite the antifungal therapy with Amphotericin B and Voriconazole, the patient did not survive.

**Conclusions:**

Despite CNS Fungal infections are mostly due to *Cryptococcus* spp., other emergent yeasts, such as *T. inkin* may be considered as a likely etiological agent. This is the first case report of CNS Trichosporonosis, where species identification was performed with rDNA sequencing.

## Background

*Trichosporon* species are basidiomycetous yeast-like fungi widely distributed in nature, predominantly found in tropical and temperate areas [1]. *Trichosporon* spp. may be found in substrates such as soil, decomposing wood, air, rivers, lakes, seawater, cheese, scarab beetles, bird droppings, bats, pigeons and cattle [1]. These organisms also are present in the human microbiota of skin and gastrointestinal tract [2]. *Trichosporon* spp. are phenotypically characterized by colonies of white or cream coloring, with dry appearance, cerebriform or radiated surface [3] and microscopically by the presence of blastoconidia, arthroconidia, pseudophyphae and true hyphae [4].

*Trichosporon* spp. are usually associated with superficial mycosis such as white *piedra* [1, 4, 5], onychomycosis [1, 5–7] interdigital and inguinocrural lesions [5, 7]. Invasive trichosporonosis is a deep-seated infection which may be observed in leukemia or lymphoma patients who developed severe neutropenia, associated with broad spectrum antibiotic therapy [2, 4]. *Trichosporon asahii* and *T. mucoides* are the most frequently isolated species in invasive trichosporonosis [4, 8]. Pubmed searches using the terms “*Trichosporon inkin*”, “invasive” and “infection” only retrieved six publications [9–14].

The establishment of fungal infection is associated with host immune conditions as well as virulence attributes of the microorganism involved. For instance, adhesion to epithelial cells [15], the production and secretion of hydrolytic enzymes such as phospholipases and hemolysins [16] and the capacity of biofilm formation [17] contribute to yeasts pathogenicity and have been demonstrated in *Trichosporon* spp.

Central nervous system (CNS) trichosporonosis is a rare clinical manifestation associated with immunocompromised patients [18]. In fact, only a few clinical cases of this medical condition have been reported in the literature [2, 4, 18–25] and none of them was due to *T. inkin*.

The gold standard diagnosis of CNS trichosporonosis is the isolation of *Trichosporon* in culture of tissue samples or cerebrospinal fluid (CSF) [1, 19]. The reference method for *Trichosporon* species identification is based on Intergenic Spacer 1 (IGS1) region of the ribosomal DNA sequencing [8, 26]. However, Matrix-Assisted Laser Desorption/Ionization Time-of-Flight (MALDI-TOF) Mass Spectrometry has been shown to be a valuable alternative to *Trichosporon* species identification [1, 27].

Despite some controversies, triazoles appear to be the best first line antifungal therapy for invasive trichosporonosis, especially when *T. asahii* is involved [1, 10, 18, 19, 27]. In vitro studies suggest that voriconazole exhibits the best antifungal activity against different *Trichosporon* species, when compared to amphotericin B and fluconazole [10]. Echinocandins are associated with high MIC values [19, 28] and indicates no action on *Trichosporon* cells. Therefore, they are not indicated in clinical practice [19, 27].

To the best of our knowledge, we describe the first case of meningoencephalitis due to *T. inkin* in a previously healthy female patient under corticosteroids regimen after having undergonea microsurgery of neuroma in Natal, Rio Grande do Norte State, Northeast Brazil. We also describe theantifungal susceptibility profiling and characterization of the expression of virulence factors in vitro of two *T. inkin* isolates sequentially obtained from the CSF of this patient and also a review of meningoencephalitis clinical cases due to *Trichosporon* spp. reported in the literature.

## Case presentation

MECM, a 49-years-old previously healthy woman, married and childless, was admitted at a private hospital in Natal City, Rio Grande do Norte State, Brazil, in June, 2014 for a microsurgery of neuroma. She used to live in a flat with a parrot who had an unknown disease that caused loss of feathers. The microsurgery was performed via the cranial middle fossa to remove a left sided acoustic neuroma. After 40 days of the procedure, she presented a predominantly and intensive occipital holocranial headache, followed by vomiting. She was managed with analgesia and prednisone 20 mg/day for 5 days. The patient also had hyporexia that was accentuated with the worsening of headache, 12 kg of weight loss, asthenia, irritability, difficulty to concentrate and rotator vertigo. She did not have a fever. On physical examination, the patient presented classic signs of irritability of meningeal inflammation.

On the 50th postoperative day, she was diagnosed with a cerebrospinal fistula in the occipital region and submitted to a surgical correction. The CSF analysis revealed 126 cells/mm^3^, composed by 63% of lymphomonocytes, 13 mg/dl of glucose levels (89 mg/dl of glycemia) and 189 mg/dL of proteins. Direct examination and CSF microbiological culturing (including common bacterial, mycobacterial and fungal procedures) did not detect any pathogen. Hemogram and biochemical examination of blood were normal. Vancomycin and ceftriaxone were prescribed for 14 days, dexamethasone, 16 mg/day, for 10 days, followed by 15 days of prednisone weaning. She was discharged with partial improvement of headache, without vomiting and presenting normal CSF. After 3 weeks, the headache intensified and vomiting returned. Prednisone 80 mg/day, for 7 days, followed by 30 days of weaning was prescribed, resulting in mild improvement of headache, but with persistent vomiting and return of rotational vertigo. Therefore, cinnarizine, esomeprazole, bromopride and paracetamol/codeine were prescribed. As no relief was obtained after 30 days, the patient was re-hospitalized and CSF analysis revealed: 245 cells/mm^3^, 88% of lymphomonocytes, 23 mg/dL of glucose levels and proteins of 324 mg/dL. Microbiological cultures for bacteria and fungi were negative. Hemogram and biochemical examination of blood were still normal. She was diagnosed again with occipital liquoric fistula and submitted to clinical treatment. She was under the same antimicrobial and corticoid regimen of the last hospitalization and was discharged with mild headache. Dexamethasone 16 mg/day, for 10 days, followed by 30 days of weaning with prednisone was prescribed. At that moment, the CSF still had 68 cells/mm^3^, with 100% of lymphomonocytes, 56 mg/dL of glucose levels and 78 mg/dL of proteins. Prednisone was prescribed for 30 days.

**Fig. 1:**
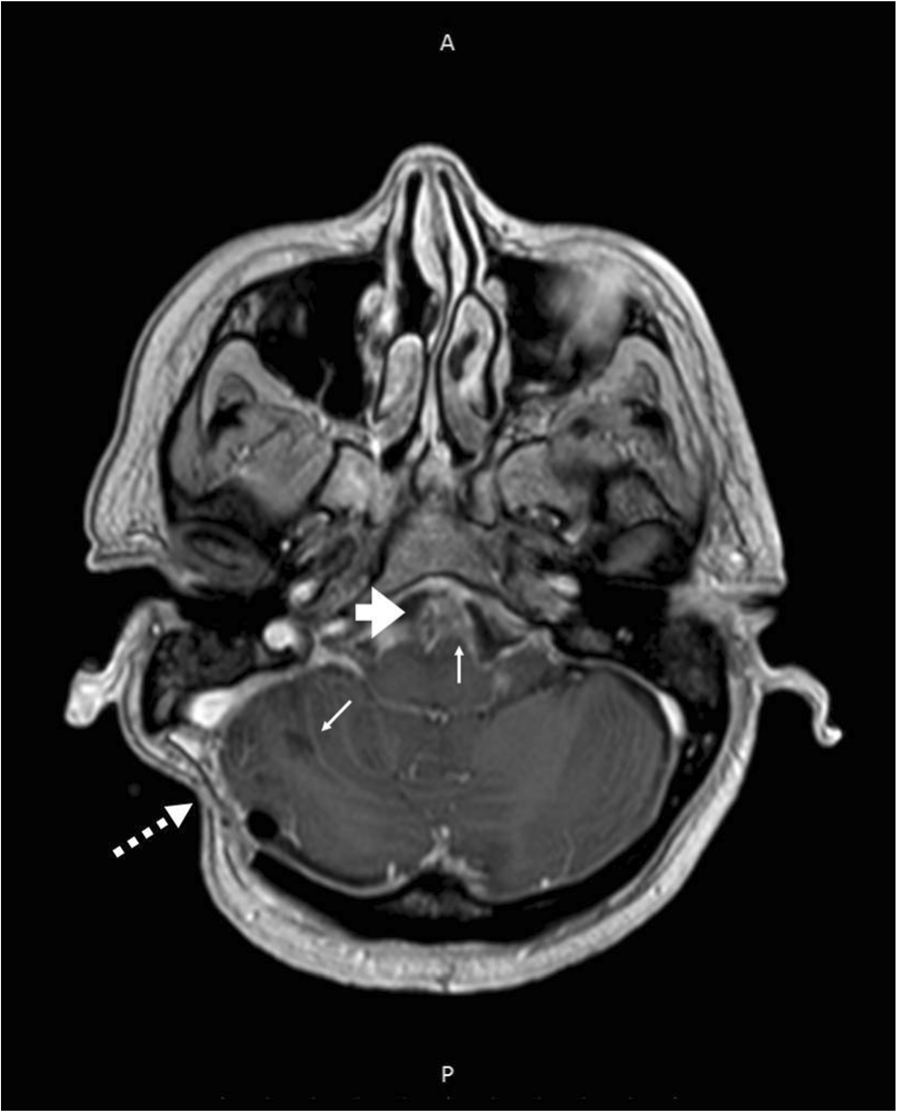
Axial Magnetic Resonance Imaging (MRI) at the posterior fossa level, showing extensive leptomeningeal enhancement near the anterior bulb contour and cerebellar folds (thin arrows). Heterogeneous material with enhancement near the pre-pontine cistern (thick arrow). Note the signs of surgical manipulation of the extra-cranial soft parts in the right occipital region (dashed arrow)

When the corticoid was discontinued, headache worsened and vomiting returned. After 5 months of the onset of the disease, a new computed tomography (CT) scan of the skull showed a CSF fistula on the same topography. She was hospitalized and submitted to a surgery to correct the fistula. She had leukocytosis on admission (16,000 leukocytes/mm^3^, with 88% segmented cells) and CSF analysis showed 280 cells/mm^3^, being 88% of lymphomonocytes cells, 12 mg/dL of glucose levels and 312 mg/dL of proteins. Bacterial and fungal cultures were negative. Empirical treatment with vancomycin and cefepime was introduced for 21 days and dexamethasone 16 mg/day for 10 days, followed by 20 days of weaning with prednisone. As the headache worsened, she was again hospitalized and submitted to surgical correction of the fistula. New CSF showed 184 cells, 63% of lymphomonocytes, 41 mg/dL of glucose levels and 285 mg/dL of proteins. Vancomycin, meropenem and dexamethasone, 10 mg/day were initiated. On the 5th day of treatment, headache remained intense and frequent vomiting. A new CT suggested hydrocephalus and the patient was submitted to a ventriculoperitoneal (VP) shunt. After 3 days of VP, the patient continued to present with vomiting and leukocytosis and the CSF pressure was above 300 mmH_2_O. She was admitted to the intensive care unit. A magnetic resonance imaging (MRI) of the skull suggested meningeal thickening, spinal cord compression at the level of C5-C6 and the alteration of the CSF signal was compatible with viral or fungal disease (Fig. 1). The initial suspicion was cryptococcosis. Liposomal amphotericin B (300 mg/day) and acyclovir therapy were empirically initiated. After several invasive procedures, broad spectrum antibiotics and corticosteroids, CSF culture showed growth of *Trichosporon* spp. After 2 weeks, another *Trichosporon* CSF positive culture was obtained. As there was progressive worsening of the clinical condition, voriconazole (200 mg/every 12 h) was added to the previous prescription. On the 20th day of hospitalization, the patient died (Table 1).

**Table 1:** Timeline of exposition to multiple risk conditions of a patient submitted to an acoustic neuroma surgery and further developed meningitis in Natal city, Rio Grande do Norte State, Northeast Brazil

Period of time	Acoustic neuroma surgery	Corticosteroids usage	Appearence of the fistula	Antibiotics usage	CSF^a^ analisys performing	Surgical fistula correction	VP^b^ shunt	Antifungal therapy	*Trichosporon* positive culture	Death
Week 1	X									
Week 6		X								
Week 8		X	X	X	X					
Week 9		X		X						
Week 13		X								
Week 17		X	X	X	X					
Week 18		X		X						
Week 19		X		X						
Week 20		X	X	X	X	X				
Week 21		X		X						
Week 22		X		X						
Week 23		X		X	X	X	X	X	X	
Week 24		X		X				X		
Week 25								X	X	
Week 26								X		X

^a^*CSF* Cerebrospinal fluid, ^b^*VP* Ventriculoperitoneal

### Culturing procedures and molecular identification of the pathogen

**Fig. 2:**
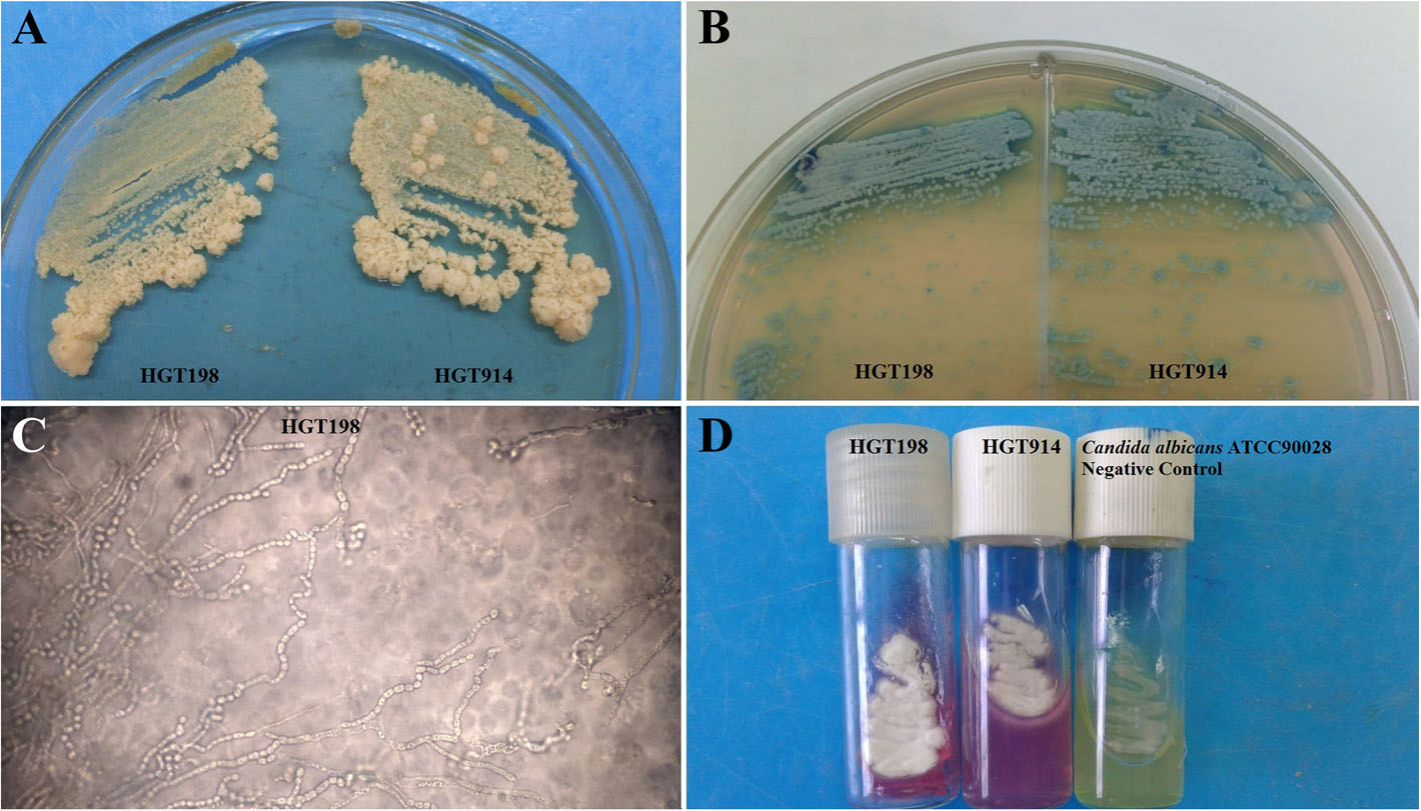
**a** Cream-colored, dull, wrinkled cerebriform colonies, after 48 h of incubation at 30 °C on Sabouraud dextrose agar. **b** Colonies with typical “dirty” grey-blue color on CHROMagar *Candida*® medium after 72 h of incubation at 35 °C. **c** Micromorphological aspects after incubation in cornmeal agar containing Tween 80 for 72 h at 30 °C, showing long true hyphae e artroconidia. **d** Urease test of yeast cells grown in Cristensen’s urea Agar containing phenol red, showing positive results after incubation at 30 °C for 72 h

The CSF was centrifuged at 2500 rpm for 10 min and the sediment was used for direct examination and culture. Direct examination was performed with India ink which revealed no encapsulated blastoconidia. The sediment of 2 CSF samples collected at different days (14th and 28th of April, 2015) were plated on Sabouraud Dextrose Agar at room temperature (28 + 2 °C) and yielded positive yeast cultures after 72 h of incubation. The two cultures were send to the Medical and Molecular Mycology Laboratory, Clinical and Toxicological Analyses Department, Federal University of Rio Grande do Norte State for further molecular identification. Of note, both colonies had a mucoid aspect. Besides, because *Cryptococcus* spp. are the main etiological fungal agents obtained from meningitis, that was the first suspicion. Yeast isolates from original cultures were plated onto CHROMagar *Candida* (CHROMagar Microbiology, Paris, France) and corn meal-Tween 80 (to induce sporulation). Surprisingly, both isolates had a macroscopic wrinkled appearance, were able to produce arthroconidia, as revealed by their micromorphology, and to hydrolyze urea (Fig. 2a to d). Therefore, they were considered to belong to the genus *Trichosporon* and named HGT198 and HGT914, respectively. Both strains were further identified by molecular techniques.

### Molecular identification

A single colony of each strain was used for DNA extraction with PrepMan Ultra sample preparation reagent (Applied Biosystems, Foster City, CA) according to the manufacturer’s instructions. Genomic DNA concentration and purity were checked with a NanoDrop instrument (Thermo Scientific; Amersham Pharmacia Biotech, Wilmington, DE, USA). Both strains were further identified by a molecular method as detailed elsewhere [29]. DNA amplification was obtained by using the primer pair TRF (5′-AGAGGCCTACCATGGTATCA-3′) and TRR (5′-TAAGACCCAATAGAGCCCTA-3′) [26]. Nucleotide sequences were submitted for BLAST analysis at the NCBI site (http://www.ncbi.nlm.nih.gov) for species identification. Only sequences deposited in GenBank showing high similarities with our query sequences and an *E*-value of lower than 10^− 5^ were used in this study. BLAST searches showed the best match with *T. inkin* (FJ153608.1), 100% identity (619 of 619 bp without gap sites) for both strains (HGT198 and HGT914). IGS1 rDNA sequences of these strains have been deposited in GenBank under accession numbers KY807052 and KY807053, respectively. Of note, both strains were considered of 100% identity, after blastn analysis (all the 641 bp compared among them), with an *E*-value of 0 and no gaps found between the two IGS1 rDNA sequences.

### Characterization of virulence factors and in vitro antifungal susceptibility

Strains HGT198 and HGT914 were evaluated according to their ability to adhere to human buccal epithelial cells, biofilm formation, hemolysins and phospholipase production by using the methods described by Zuza-Alves [30]. DNAse production was determined according to Montoya [16]. Both strains did not produce phospholipase or DNAse. However, they showed high biofilm formation capability as compared to *C. albicans* ATCC90028 and *T. asahii* CBS2630 and similar levels of hemolysin production of the two reference strains. In addition, they were able to adhere to epithelial cells to the same extension of *T. asahii* reference strain (Table 2).

**Table 2:** Evaluation of attributes of virulence factors in vitro of *Trichosporon inkin* isolates HGT198 and HGT914 obtained from a patient submitted to an acoustic neuroma surgery and further developed meningitis in Natal city, Rio Grande do Norte State, Northeast Brazil

	N^o^ of *T. inkin* cells adhered to 150 HBEC	Hemolytic index (*HI*)	Biofilm formation (OD_595nm_)
*Candida albicans* ATCC90028	179.8 ± 2.05	0.63 ± 0.01	0.24 ± 0.03
*Trichosporon asahii* CBS2630	34.3 ± 1.70	0.74 ± 0.01	0.40 ± 0.01
*Trichosporon inkin* HGT198	36.7 ± 1.50^a^	0.68 ± 0.01^a; b, c^	0.77 ± 0.05^a; b, c^
*Trichosporon inkin* HGT914	37.3 ± 1.50^a^	0.56 ± 0.01^a; b, c^	1.04 ± 0.01^a; b, c^

*NT* Not Tested

^a^Statistically significant different from *Candida albicans* ATCC90028

^b^Statistically significant different from *Trichosporon asahii* CBS2630

^c^Statistically significant difference between HGT198 and HGT198

Both strains were tested against fluconazole, itraconazole and amphotericin Bby using the CLSI protocol [31–33]. As illustrated on Table 3, they exhibited very low MIC values against all antifungal drugs tested.

**Table 3:** Determination of antifungal susceptibility testing of *Trichosporon inkin* isolates HGT198 and HGT914 obtained from a patient submitted to an acoustic neuroma surgery and further developed meningitis in Natal city, Rio Grande do Norte State, Northeast Brazil

Strain	Fluconazole (24 h)	Itraconazole (48 h)	Amphotericin B (48 h)
*Candida parapsilosis* ATCC 22019	1 μg/mL	NT	NT
*Candida krusei* ATCC6258	16 μg/mL	NT	NT
*Trichosporon inkin* HGT-198	0.5 μg/mL	0.062 μg/mL	0.5 μg/mL
*Trichosporon inkin* HGT-914	0.5 μg/mL	0.062 μg/mL	0.5 μg/mL

## Discussion and conclusions

*Trichosporon* species are present in the environment and may belong to the human microbiota butmay associated with both superficial and deep infections [1, 18]. Infections associated with CNS due *Trichosporon* are rare in immunocompromised patients and extremely rare in immunocompetent patients [20].

The number of cases of invasive trichosporonosis reported worldwide may be still considered restrict once a limited number of publications may be found in the literature [27]. A recent epidemiological study conducted by De Almeida Júnior et al. [27] reviewing global cases of invasive trichosporonosis retrieved from PubMed from 1994 and 2015 only found a total of 203 cases of invasive trichosporonosis, where *T. asahii* accounted for 95 (47.6%) of all cases [27].

The first case of SNC infection due *Trichosporon* was described by Watson and Kallichurum in 1970 [25] in a 39-year-old African woman diagnosed with underlying bronchial adenocarcinoma and brain abscess due *T. cutaneum* [25]. It is important to mention that no molecular techniques were available at this time. Therefore, this could have led to an unreliable species identification. Since then, only other few case reports of *Trichosporon* meningitis have been published in the literature, but none of them was due to *T. inkin* (Table 4).

**Table 4:** Systematic review of *Trichosporonosis* meningitis cases published in the literature from to 1970 to 2018

Country	Sex/Age	Diagnosis/Underlying diseases	Species isolated	Clinical Sample	Treatment/outcome	Year/Reference
Brazil			*Trichosporon inkin*	CSF		Present study
Singapore	F/50	Disseminated trichosporonosis/Aplastic Anemia	*T. asahii*	CSF, Blood	AMB, VOR, ITR, POS/ Survived	2016 [24]
India	M/18	Chronic meningo-ventriculitis and intraventricular fungal ball/immunocompetent	*T. asahii*	Intraventricular biopsy and CSF	AMB/died	2015 [20]
Iran	M/34	Brain abscess/ autoimmune hepatitis, hypothyroidism	*T. asahii*	Brain abscess	Surgical resection, AMB and ITC/ survived	2012 [18]
India	NI	Meningitis/Acquired Immunodeficiency Syndrome (AIDS)	*Trichosporon* sp.	CSF	AMB, FLU, survived	2012 [19]
Jamaica	F/44	Meningitis and cerebral abscess/diabetes, burns	*T. asahii*	Facial wounds, sputum, and a meningeal swab	None/died	2011 [4]
Taiwan	NI	Meningitis/NI	*T. montevideense*	CSF	NI	2009 [2]
India	M/18	Disseminated trichosporonosis/Imunnocompetent	*T. asahii*	CSF	FLU/survived	2007 [22]
India	F/36	Chronic meningitis/ Chronic back pain after fall	*T. beigelii*	CSF	None/died	1995 [21]
Belgium	M/15	Meningitis/acute lymphocytic leukaemia	*T. beigelii*	CSF	AMB, FC and FLU/died	1990 [23]
South Africa	F/39	Brain abscess/adenocarcinoma	*T. cutaneum*	Brain lesions	None/died	1970 [25]

The geographic distribution of invasive trichosporonosis with CNS complications is higher in Asia (seven cases) [2, 18–22, 24]. There are cases described in Europe [23], Africa [25] and Central America (one case each) [4]. Clinical series with patients’ demographic data and underlying conditions may be found in Table 4.

There is only a single study reporting *Trichosporon* meningitis in an immunocompetent patient [22]. In this study, Rastoji et al. [22] reported a case of invasive trichosporonosis with CNS complications in a 18-years-old male, who presented fever, chills and rigor associated with headache, nausea, vomiting and altered sensorium. *T. asahii* was isolated from CSF and sputum of this patient [22].

Several clinical conditions are considered to be risk factors for invasive trichosporonosis including the history of intensive chemotherapy, high dose of corticosteroids, burns, neutropenia, broad spectrum antibiotics usage and hematological malignancies [8, 20, 27]. In fact, the patient described in this study was previously immunocompetent but further submitted to long periods of corticosteroids and broad-spectrum antibiotics, besides suffering the invasive medical procedure to remove an acoustic neuroma. Of note, the CSF fistula probably had an important role for local *Trichosporon* contamination. In addition, the fact that she had a parrot could have led to yeasts skin colonization, once these birds may harbor *Trichosporon* in their gastrointestinal tract [34]. A limitation of our study is that we did not perform her parrot’s droppings culture, trying to isolate *Trichosporon* and further checked with molecular techniques to determine the probable source of infection.

Our isolates were as able to adhere to epithelial cells as *T. asahii* CBS2630 that is considered the most virulent and frequently isolated species of the genus *Trichosporon*, as demonstrated by virulence studies with *Galleria mellonella* and murine models of systemic infection [35]. Of note, all *Trichosporon* isolates were less adherent than *Candida albicans* ATCC90028. This was expected, because *C. albicans* is widely recognized as the more adherent *Candida* species [36], but it was used as a reference strain for adhesion assay.

Both isolates (HGT198 and HGT914) had stronger hemolytic activities than reference strain *T. asahii* CBS2630. Our isolates were considered highly hemolytic according with the criteria established by Montoya et al. [16]. These authors reported a single strain of *T. asahii* with strong hemolytic activity, while the other 38 isolates did not produce this enzyme. These finding reinforce virulence attributes properties of the isolates from the present study that may have influenced clinical outcome.

Biofilm formation by microorganisms has gained attention within clinical practice because of its ability to increase mortality in patients with systemic infections by yeasts [37]. Studies on biofilm formation in *Trichosporon* have increased in recent years, since this yeast has been considered the second most common etiological agent of systemic infection in patients with hematological malignancies [38]. In the presence of biofilm, structured microbial communities remain embedded within an extracellular polymeric substance, where *Trichosporon* spp. show significantly greater resistance to antifungals, with MICs ranging from 128 to 1.024 μg/mL for Amphotericin B and 512–1.024 μg/mL for Fluconazole, Itraconazole, and Voriconazole [38]. Our isolates showed an optical density of 595 nm (which reflects biofilm biomass stained by crystal violet) at least two-fold higher than the readings found for *T. asahii* CBS6030. Of note, there is a trend of increased virulence attributes expression over time when both strains isolated 2 weeks apart were compared (Table 1). This phenomenon is observed for both hemolysins and biofilm production. This may reflect strain adaptation to the host in the progress of infection.

Our strains were not resistant to any of the antifungal drugs tested. In addition, MICs obtained from *T. inkin* HGT198 and HGT914 did not increase over time. This observation indicates that probably there was not induced antifungal resistance for the strains recovered within 2 weeks of difference. It is important to emphasize that antifungal susceptibility testing was only possible to be performed after patient’s death and was not used to drive antifungal therapy.

There are some limitations about the interpretations of MIC values in our *Trichosporon* isolates. First of all, there are still no breakpoints established from Clinical & Laboratory Standards Institute (CLSI) and European Committee on Antimicrobial Susceptibility Testing (EUCAST) Antifungal Susceptibility testing to *Trichosporon* species [27], and second, there are no studies which determined MICs of *T. inkin* strains isolated from patients with meningoencephalitis.

Susceptibility testing to the antifungal drugs Amphotericin B, Itraconazole and Fluconazole were performed by Taj-Aldeen et al. [39] with three *T. inkin* clinical isolates obtained from urine and white *piedra*. MIC range was 1–4 μg/mL (Amphotericin B), 0.25–4 μg/mL (Fluconazole) and 0.013–0.125 μg/mL (Itraconazole). *T. inkin* clinical isolates from bone, urine, skin, subcutaneous abscess, peritoneal liquid and blood susceptibility profiling was determined and MIC range was 0.06–1 μg/mL (Amphotericin B), 1–32 μg/mL (Fluconazole) and 0.06–2 μg/mL (Itraconazole) [40]. In the present study, our *T. inkin* isolates had lower MIC values when compared with other publications [39, 40]. We observed that Itraconazole exhibited better in vitro effect against *T. inkin* isolates compared to Fluconazole and Amphotericin B and this finding was also observed by other studies involving *T. inkin* [39, 40] and other *Trichosporon* species [2, 35].

In conclusion, we may say this is the first case report of meningitis caused by *T. inkin* reported in the literature. Our female previously healthy patient was under corticosteroids and antibiotics therapy for a few months. In addition, she was submitted to an invasive procedure to remove a left sided acoustic neuroma and further developed a cerebrospinal fistula. All those factors are consolidated risk conditions for the infection caused by *T inkin*. The certainty of the invasive infection by *T. inkin* was based on the isolation of the pathogen along two different occasions together with brain images and cytological findings suggestive of meningitis. Both strains showed strong ability to express virulence factors in vitro. These findings together with patient’s immunological status may have been crucial for the clinical outcome, because the strains were apparently not resistant to the antifungal drugs prescribed during her period of hospitalization. The physicians main suspicion was cryptococcal meningitis. Corroborating this idea, the strains obtained from CNF presented mucoid aspect in the primary isolation. However, the sending of these isolates to a reference Mycology center for accurate phenotypic and molecular identification revealed that the meningitis caused by *Trichsporon inkin*.

## Abbreviations

ATCC: American Type Culture Collection
BLAST: Basic Local Alignment Search Tool
CBS: Centraalbureauvoor Schimmel cultures
CLSI: The Clinical & Laboratory Standards Institute
CNS: Central nervous system
CSF: Cerebrospinal fluid
CT: Computed tomography
Fig.: Figure
IGS1: Intergenic Spacer 1
MALDI-TOF: Matrix-Assisted Laser Desorption/Ionization Time-of-Flight
MIC: Minimal Inhibitory Concentration
MRI: Magnetic Resonance Imaging
rpm: Revolutions per minute
VP: Ventriculoperitoneal
